# Virology and Molecular Pathogenesis of Coronavirus Disease 2019: An Update

**DOI:** 10.5152/eurasianjmed.2022.21133

**Published:** 2022-10-01

**Authors:** Kalpana Panati, Lokesh V Timmana, Venkatramana Reddy AT, Rajeswara Reddy Saddala, Venkata Ramireddy Narala

**Affiliations:** 1 Department of Biotechnology, Government College for Men, Kadapa, A.P, India; 2 Department of Zoology, Yogi Vemana University, Kadapa, A.P, India; 3 Department of Biotechnology, Dravidian University, Kuppam, A.P, India

**Keywords:** COVID-19, histology, pneumonia, SARS-CoV2, severe acute respiratory syndrome, virology

## Abstract

The pandemic coronavirus disease 2019 outbreak’s causative agent was identified as severe acute respiratory syndrome coronavirus 2. It is a positive-sense single-stranded RNA virus with a ~30 kb size genome that belongs to the Nidovirales. Molecular analysis revealed that severe acute respiratory syndrome coronavirus 2 is a variant of severe acute respiratory syndrome coronavirus and Middle East respiratory syndrome coronavirus with some sequence similarity. The confirmed cases and death toll are high in severe acute respiratory syndrome coronavirus 2 compared to severe acute respiratory syndrome coronavirus and the estimated R_0_ is >1. The data on pathological findings on severe acute respiratory syndrome coronavirus 2 are scarce and present treatment management is based on symptoms that are similar to severe acute respiratory syndrome coronavirus. In this review, we have discussed the transmission, viral replication, and cytokine storm and highlighted the recent pathological findings of coronavirus disease 2019. The reported severe acute respiratory syndrome coronavirus 2 pathological findings were similar to that of severe acute respiratory syndrome coronavirus. Though these findings help notify the clinical course of the disease, it warrants further in vivo and ex vivo studies with larger samples obtained from the coronavirus disease 2019 patients.

**Main Points**

- Severe acute respiratory syndrome coronavirus 2 uses multi-subunit machinery for replication/transcription. Understanding this machinery will be critical to develop strategies to prevent the entry of the virus.
- The main pathological findings in the lungs include bilateral diffuse alveolar damage with cellular fibromyxoid exudates, pulmonary edema, and hyaline membrane formation.
- The data suggest an increased risk of thrombosis in severe coronavirus disease 2019 (COVID-19) patients.
- Enormous variation in infectivity and pathological findings were observed in the first and second waves of COVID-19.
- Well-designed studies of different isolates on mutagenicity, tissue tropism, replication, and pathogenesis of virus are highly warranted.

## Introduction

Coronavirus disease 2019 (COVID-19) is an emerging, rapidly spreading pandemic initially reported in China. As the second wave continues to hit, over 169 million COVID-19 cases and 3.53 million deaths have been reported globally indicating a continuous threat to society. The animal source and the intermediate reservoir of the current pandemic are still unknown and it was reported that severe acute respiratory syndrome coronavirus 2 (SARS-CoV2), which belongs to betacoronaviruses, was the causative agent of the COVID-19 outbreak. The viral genome of SARS-CoV2 was found similar to SARS-CoV and Middle East respiratory syndrome (MERS) CoV to some extent^1^ and the pathological features greatly resemble those seen in SARS and MERS coronavirus infection.^2,3^ The spike protein plays a major role in binding to the receptor on the host cell, membrane fusion,^4^ and transmission capacity.^5^ Mutational heterogeneity was also observed in spike protein among various virus variants isolated across the globe that may influence host infectivity rate or susceptibility.^6^ Amino acid sequence of the spike protein of SARS-CoV2 showed more similarity (76.47%) with SARS-CoV.^7^ Despite some variations in COVID-19 virus receptor binding domain, it was suggested COVID-19 virus might use angiotensin-converting enzyme 2 (ACE2) as a cell membrane receptor.^1^ As there is no specific antiviral treatment available for COVID-19, the fatality rate is continuously increasing in severe cases.^8^ This review highlighted and discussed the recent updates on virology, transmission, pathogenesis, and pathological findings of COVID-19 infection.

### Coronaviruses—Complex Genome Organization

Severe acute respiratory syndrome coronavirus 2 belongs to the Nidovirales order of coronaviruses containing enveloped, non-segmented positive-sense RNA genome of ~30 kb and is found to be the causative agent of COVID-19. Coronaviruses quickly adapt to the new environment and alter host range efficiently, thereby giving a constant and long-term health threat to humans.^9^ Coronaviruses’ genome shows a complex organization consisting of a 5’ cap structure and a 3’ poly (A) tail (similar to eukaryotes) to facilitate the immediate translation of replicase polyproteins. Replicase genes occupy two-thirds of the genome which code for nonstructural proteins and the remaining one-third of the genome (~10 kb) code for structural proteins and proteins required for pathogenesis indicating meticulous regulation of transcription and translation of the viral genome. The genome shows stringent control over the expression of each structural or accessory gene, additionally by having transcriptional regulatory sequences, 5’ untranslated region (UTR) at the beginning and 3’ UTR at each gene. Moreover, it expresses many genes by ribosomal frameshifting.^10^ It is unknown why coronaviruses utilize this mechanism to control protein expression.

### Spill over to Humans

It is well known that the mutations in the viral genome enable the virus to cross the infective host species.^10^ According to Angeletti et al^11^ after analyzing the transmembrane helical segments, they found at position 723, serine was present instead of glycine; at position 1010, proline was present instead of isoleucine in the ORF1ab encoded nsp2 and nsp3 in SARS-CoV2 when compared to SARS-CoV and Bat SARS-like CoV. These mutations in nsp2 protein could be transmitted from bats and infect humans with a highly contagious mode.^11^

### SARS-CoV2

At the end of 2019, pneumonia patients with an unknown cause were reported in Wuhan, China. Clinicians categorized this disease as virus-induced pneumonia based on clinical symptoms, blood tests, and chest radiographs. The virus was isolated from the patients, cultured, and nucleic acid sequencing was completed. Using this sequence information, the evolutionary tree analysis revealed that novel β-coronavirus belongs to the sarbecovirus subgenus of the Coronaviridae family.^12^ The novel coronavirus (SARS-CoV2) showed 86.9% sequence identity with bat SARS-like CoV genome and organized into a 5’ UTR, replicase complex (orf1ab), spike (S) gene, envelope (E) gene, membrane (M) gene, nucleocapsid (N) gene, 3’ UTR, and several unidentified nonstructural open reading frame (ORF) and it is distinct from SARS-CoV and MERS-CoV.^12^ However, 94.4% sequence identity was found between the replicase domains in ORF1ab of SARS-CoV2 and SARS-CoV.^13^

### Transmission

At present, direct contact, respiratory droplets, and fomites are considered as means of transmission. Though the SARS-CoV2 was found in the urine and stools of patients, the fecal–oral route of transmission needs to be investigated.^14^ Further, no study reported the intrauterine vertical transmission of SARS-CoV2 to date.^15,16^ The incubation period was estimated as 14 days which was longer than the SARS-CoV. The asymptomatic persons may transmit the virus if they carry a high viral load. The average number of persons who can get the disease from 1 contagious person (R_0_) for COVID-19 is estimated as greater than 1 indicating continuous transmission.^14^

## Pathogenesis

### Viral Entry into the Host Cell

Usually, in coronaviruses, homotrimers of S protein make spike-like structures on the surface of virus^17^ and it mediates the attachment to the host cell receptor.^18^ Apart from mediating the virus entry into the host cell, spike protein acts as a critical determinant of virus–host range, tissue tropism, and inducing host immune responses.^4^ Among the 3 domains of spike protein (ectodomain, transmembrane anchor, and intracellular tail), the ectodomain of S protein contains receptor-binding domain (S1) and membrane fusion domain (S2) which were cleaved by host factors.^19^ The S1-C terminal domains are responsible for receptor recognition. Severe acute respiratory syndrome coronavirus 2, similar to the SARS-CoV, uses ACE2 as a receptor to enter the human cells.^13^ Angiotensin-converting enzyme 2 is a type I membrane protein known to express in lungs, heart, kidneys, blood vessels, and intestine. In addition to modification of angiotensin, ACE2 also provides a binding site for S protein of coronaviruses.^20^ S1-N terminal domain interacts with glycans and facilitates binding of S1 to host cell receptor. It destabilizes the pre-fusion trimer, shedding of S1 subunit, and forms a highly stable post-fusion S2 subunit.^21^ Then, S2 fuses the host and viral membranes using the energy released during the conformational transition of S2.^4^ A recent study also provided biophysical and structural evidence to show that the spike protein of SARS-CoV2 binds to ACE2 with higher affinity than the SARS-CoV.^22^ As evidenced, the spike protein plays an important role in the entry of virus; this protein may be a therapeutic target to develop inhibitors.^23^ It was also suggested that the ACE polymorphism may have a combined effect on infectivity, recovery, and pathogenesis of COVID-19.^24^

### Viral Replication

**Figure 1.**
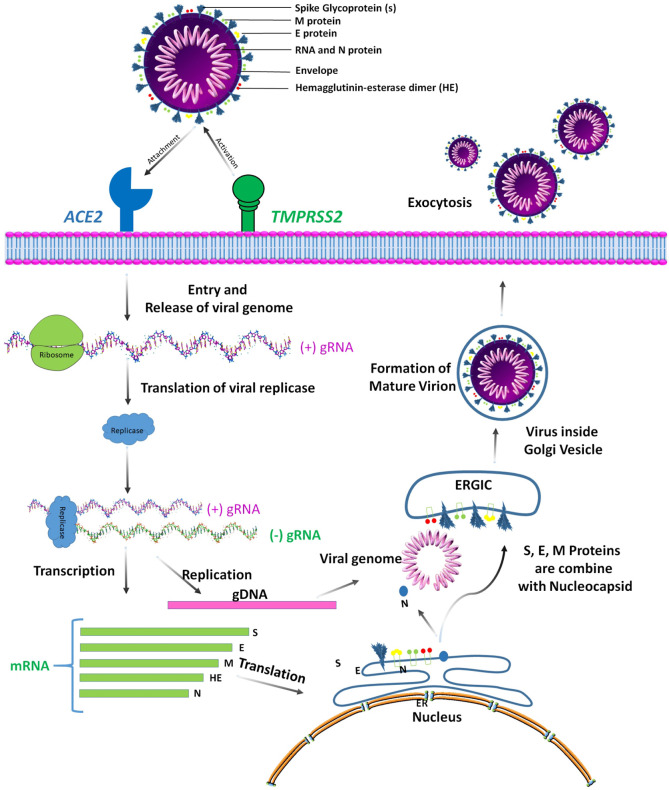
The life cycle of SARS-CoV2 in the host cell. SARS-CoV2, severe acute respiratory syndrome coronavirus 2.

Coronaviruses use multi-subunit machinery for replication/transcription. The virus enters the target cell through an endosomal pathway. Spike glycoprotein is activated by transmembrane protease serine 2 (TMPRSS2) and binds to the ACE2 receptor. After the viral entry into the cytoplasm, the positive sense genomic RNA (+gRNA) is released and acts as a template for the formation replicase–transcriptase complex (RTC). Further, this complex produces negative-sense RNA [(−) RNA]. These RNA molecules are utilized for the synthesis of +gRNA of the virions. Virions are then released from the infected cell through exocytosis (Figure 1). The replicase gene encodes 2 ORFs (rep1a and rep1b) and produces 2 polyproteins (pp1a and pp1ab) by using host machinery. Coronaviruses precisely maintain the ratio of these 2 proteins by using a slippery sequence (5’-UUUAAAC-3’) and a pseudoknot which cause ribosomal frameshifting of ORF.^10^ A nonstructural protein 12 (nsp12) plays a central role by acting as RNA-dependent RNA polymerase in catalyzing the synthesis of the viral genome with the help of other nsps by forming the RTC. Virus-specific nsp12 could also be targeted to develop inhibitors to reduce the nsp12 activity. Then the viral genomes and the viral structural proteins were processed as mature virions through the endoplasmic reticulum–Golgi intermediate compartment where encapsulation with nucleocapsid protein occurs (Figure 1).^25^ Nucleocapsid protein was highly phosphorylated probably to enhance the affinity for viral RNA than the host RNA while packing.^10^ In many coronaviruses, spike protein does not assemble into virions instead it transits to the cell surface and mediates the formation of cell–cell fusion between infected and nearby uninfected cells. It results in giant, multinucleated cells which facilitate viral spread within the infected host without getting neutralized by virus-specific antibodies.^10^ This could be one of the pathways used by the virus to evade recognition by the host immune system.

### Host Immune Response

The host’s immune response mounted against SARS-CoV2 is currently unknown as no experimental reports are available on SARS-CoV2 antigen presentation to the immune cells. However, the similarity between SARS-CoV and SARS-CoV2 gives us some hints to predict the host response, thereby understanding the SARS-CoV2 antigen presentation which certainly helps in designing the specific drug targets. Generally, once the virus enters the host cell, viral antigens will be processed and presented to antigen-presenting cells by major histocompatibility complex (MHC). Then specific T- and B-cells are activated and produce an immune response. Viral antigens were mainly presented by MHC-I, but MHC-II also contributes to the presentation.^26^

### Cytokine Storm

In SARS and MERS patients, it has been shown that increased levels of proinflammatory cytokines and chemokines were associated with lung injury and pulmonary inflammation.^27,28^ A recent report showed decreased CD4 and CD8 T cells in the peripheral blood of SARS-CoV2-infected patients.^29^ In addition to lymphopenia, higher plasma levels of cytokines and chemokines (IL-2, IL-7, IL-10, TNFα, MCP-1, MIP-1A, G-CSF, and IP-10) were observed in the intensive care unit (ICU) patients compared to non-ICU COVID-19 patients.^30^ Other plasma cytokine and chemokine (IFNγ, IL1β, IL1RA, IL7, IL8, IL9, IL10, basic FGF, GCSF, GMCSF, IP10, MCP1, MIP1A, MIP1B, PDGF, TNFα, and VEGF) levels were higher in COVID-19 patients compared to healthy subjects.^30^ In an ex vivo study, human lung tissue was infected with SARS-CoV or SARS-CoV2 and the infectivity, cell tropism, and proinflammatory cytokine/chemokine levels were compared.^31^ Their results indicated that SARS-CoV2 had more infectivity rate (3.2-folds) and similar cell tropism in targeting (type-1, type-2 pneumocytes, and alveolar macrophages) compared to SARS-CoV. Whereas, SARS-CoV2 infection upregulated only (38.46%) inflammatory mediators compared to SARS-CoV (84.62%) despite a higher infection rate.^31^ These results may provide some clues to the infectivity and pathogenesis of SARS-CoV2. It has also been noted that in SARS-CoV2, patients show high levels of IL1β, IFNγ, IP10, and MCP1 which may lead to activation of Th1 response, whereas SARS-CoV2 infection also showed increased secretion of IL4 and IL10 which is associated with Th2 response which is not seen in SARS-CoV infection.^27^ More studies are required to further understand Th1 and Th2 response mechanisms in SARS-CoV2 immune response. This “cytokine storm” may lead to viral sepsis, inflammation-induced lung injury, pneumonitis, acute respiratory distress syndrome (ARDS), respiratory failure and ultimately death.^32^

### Clinical Symptoms

The signs and symptoms of COVID-19 may appear 2-14 days after the exposure. The common symptoms of COVID-19 may include fever, tiredness, dry cough, and shortness of breath. Some patients may experience other symptoms like body pains, nasal congestion, runny nose, sore throat, and diarrhea.^33^ Some people experienced the loss of smell and taste.^34^ Generally, around 80% of people will recover from the disease without special treatment, whereas 1 out of 6 people suffering from other comorbidities become seriously ill and require ICU admission.^35^ Many COVID-19 patients (~50%) are afebrile and can still spread the SARS-CoV2 efficiently.^36,37^

### Pathological Findings of COVID-19 Patients

**Figure 2.**
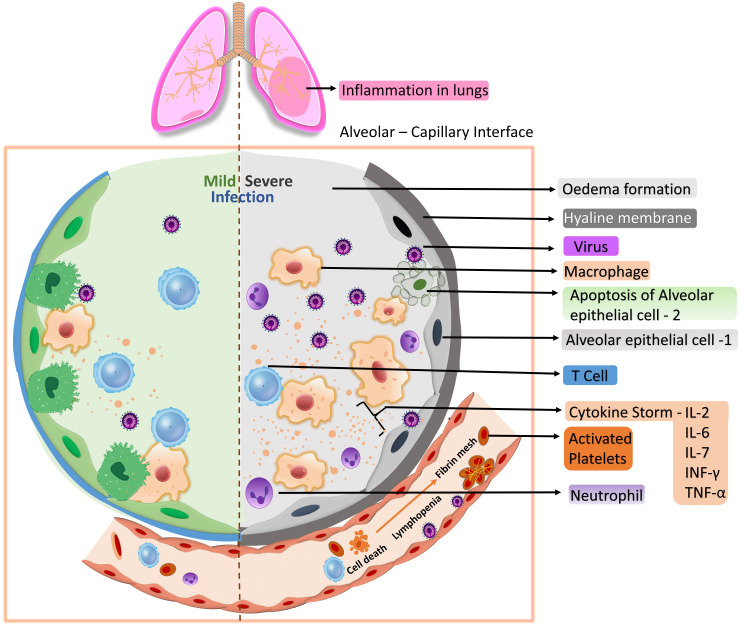
Pathogenesis of SARS-CoV2 virus in host respiratory system. SARS-CoV2, severe acute respiratory syndrome coronavirus 2.

As explained in the above section, SARS-CoV2 may pass through the mucus membranes of the nasal and larynx, and then it may enter deep into the lungs via respiratory tracts. Generally, the epithelial cell lining of the tracts becomes damaged and leads to inflammation (Figure 2). The median time from symptom onset to ARDS was about 8 days.^38^ Depending on the immune functions of the patients, if it is effective, the acute pneumonia phase can be suppressed and enter into the recovery phase. In severe cases with impaired immune function, the infection goes deep into the airways and it might result in death due to massive alveolar cell damage and progressive respiratory failure.^30^ The pathogenic mechanisms of pneumonia are complex and involve many inflammatory and lung cells. The data on pathological changes on the COVID-19 patients are scarce and more patient-driven research will have to explain many clinical aspects underlying disease progression.

Recently, a few case reports were published on the pathological findings of COVID-19 associated with ARDS by using post-mortem biopsies of lungs.^29,39^ Histological analysis of the lung showed bilateral diffuse alveolar damage (DAD) with cellular fibromyxoid exudates. Pulmonary edema and hyaline membrane formation with desquamation of pneumocytes in that lung were also found (Figure 2). Diffuse alveolar damage-dominant patterns in patients with less than 10 days of duration and acute, fibrinous, and organizing pneumonia-dominant pattern with disease duration of more than 20 days in post-mortem biopsies and autopsies of COVID-19 patients were also observed.^40^ Infiltrations of mononuclear inflammatory cells dominated by lymphocytes were observed in the lungs. Cells with multinucleated, atypical enlarged pneumocytes with large nuclei, amphophilic granular cytoplasm were found in the intra alveolar space. The above observed pathological findings were very similar to the findings of SARS-CoV and MERS CoV.^2,3^ X-ray photography showed a rapid progression of pneumonia. Peripheral blood analysis revealed that CD4 and CD8 T cells were greatly reduced, whereas their status is hyperactivated in COVID-19 patients.^29^ These results suggest severe immune injury in these patients.

In another case report of a man in his early 70s with a history of diabetes and hypertension, similar pathological findings were found in the lungs. The histopathologic examination of the lung showed DAD, organizing phase, denuded alveolar lining cells with reactive type 2 pneumocyte hyperplasia. Intra-alveolar fibrinous exudates, loose interstitial fibrosis and chronic inflammatory infiltration, and the presence of intra-alveolar organizing fibrin seen in most foci were observed. Prominent expression of Rp3NP protein (which is highly conserved between SARS-CoV and SARS-CoV2) of SARS-CoV2 on alveolar epithelial cells including damaged, desquamated cells within the alveolar space were observed with immunostaining of lung sections.^39^ In another case study report of a pregnant woman in her late 20s, histological examination showed increased wall thickness of alveolar tissue and hyaline membrane formation consistent with ARDS. Several viral cytopathic effects such as multinucleated cells and nuclear atypia were observed. The presence of infiltration of inflammatory cells was identified as lymphocytes and macrophages.^41^ Compared to mild cases, in severe cases, apoptosis of epithelial cells, edema formation, thickening of the hyaline membrane, cytokine storm (IL-2, IL-6, IL-7, IFN-γ, and TNF-α) due to hyperimmune response, and fibrin mesh formation were observed. The detailed pulmonary pathological features were represented schematically in Figure 2. Besides respiratory failure, from autopsy, imaging, and laboratory studies, it is evident that an increased risk of thrombosis could lead to increased death in COVID-19 patients. One of the reasons for the COVID-19-associated disastrous microvascular injury syndrome could be associated with the procoagulant state and activation of complement pathways.^42^ The detailed description, update on coagulopathy, thrombosis, and rationale for anticoagulation has been discussed elsewhere.^43,44^ It was suggested that the finding of elevated cardiac biomarkers (troponin, natriuretic peptides, and d-dimer) could help in identifying the severe cases of COVID-19 and further management of treatment with cardiovascular co-morbidities.^45,46^ Recently, an opportunistic fungal infection mucormycosis in severe COVID-19 disease has been identified and it poses a new threat to the recovered patients.^47,48^

### Treatment

Unfortunately, there is no single medication approved for the treatment of COVID-19 to date. However, various strategies have been developed to stop/prevent/reduce the replication of SARS-CoV2 in patients, such as antiviral drugs (remdesivir and lopinavir), anti-malarial drugs, nucleoside analogs, monoclonal antibodies with little or no success.^49^ Neutralization of SARS-CoV2 with monoclonal antibody cocktail is the latest strategy for COVID-19 treatment. Recently, the early results of a study showed a promising response toward preventing the infection of SARS-CoV2 virus in patients. Casirivimab–imdevimab and bamlanivimab are the monoclonal antibodies that received emergency use authorization by the United States Food and Drug Administration to treat high-risk COVID patients. This study showed that none of the 25 solid organ transplant recipients treated with casirivimab–imdevimab required hospitalization due to COVID-19.^50^ It may reduce the mortality rate due to COVID-19; however, further well-designed studies are warranted. Various types of vaccines developed for SARS-CoV2 were reviewed elsewhere.^51^

## Conclusions

Based on the above discussions, as COVID-19 has R_0_ of more than 1, though the effective vaccines are available, finding efficient therapy is the utmost urgent task to save the lives of severe COVID-19 patients. More studies on epidemiological and pathobiological features of COVID-19 are urgently needed to get information that ultimately benefits viral control and prevention. The current pathological findings from case studies offer new insights into pathogenesis. It could definitely help clinicians formulate an apt therapeutic strategy for similar cases to reduce fatality. There is a need to develop sophisticated and suitable in vitro and animal models to clearly understand the infectivity and pathophysiology of COVID-19.
